# Characterization of Extracellular Vesicles from Cilia and Epithelial Cells of Ductuli Efferentes in a Turtle (*Pelodiscus sinensis*)

**DOI:** 10.3390/ani9110888

**Published:** 2019-11-01

**Authors:** Imran Tarique, Yifei Liu, Xuebing Bai, Abdul Haseeb, Ping Yang, Yufei Huang, Wenjia Qu, Ruizhi Wu, Waseem Ali Vistro, Quisheng Chen

**Affiliations:** MOE Joint International Research Laboratory of Animal Health and Food Safety, College of Veterinary Medicine, Nanjing Agricultural University, Nanjing 210095, China; samoo_imran88@hotmail.com (I.T.); 2017807120@njau.edu.cn (Y.L.); 2016107003@njau.edu.cn (X.B.); 2016207037@njau.edu.cn (A.H.); yangping@njau.edu.cn (P.Y.); 2017207007@njau.edu.cn (Y.H.); 2018807169@njau.edu.cn (W.Q.); 2018107004@njau.edu.cn (R.W.); 2017207039@njau.edu.cn (W.A.V.)

**Keywords:** extracellular vesicles, exosomes, apical blebs, cilia, efferent duct, turtle

## Abstract

**Simple Summary:**

Ductuli efferent is a conduit passage of spermatozoa from rete testis to the epididymis. After spermiation, various extracellular vesicles are released by epithelial cells to assist and transfer micro-molecules for spermatozoa maturation. To date, there is a lack of data in ductuli efferent. In this study, we investigate the distribution, classification, and source of multivesicular bodies (MVBs) and their extracellular vesicles (EVs) in the epithelia of efferent ducts in a turtle by using light and transmission electron microscopy. Immunohistochemistry of CD63 (Cluster of differentiation 63) and ultrastructure demonstrates that ciliated and non-ciliated cells of ductuli efferent possess well developed exocytic systems. Ciliated cells and non-ciliated cells secrete extracellular vesicles via ciliary blebs and apical blebs, respectively. Results also showed numerous extracellular vesicles between these cells and indicate basolateral secretion. Moreover, these extracellular vesicles are associated with spermatozoa in the lumens of ductuli efferentes. Collectively, the present study provides cytological evidence that the ductuli efferentes (DE) epithelium secretes EVs to the lumen by (1) apical blebs, (2) ciliary blebs, and (3) from the basolateral region. Characterization and cellular distribution of these extracellular vesicles in the ductuli efferent of turtles may provide a study model to further investigate the transferring of micro-molecules via extracellular vesicles to the spermatozoa.

**Abstract:**

The ductuli efferentes (DE) form a transit passage for the passage of spermatozoa from the rete testis to the epididymis. After spermiation, various epithelial secretory proteins are transferred via extracellular vesicles (EVs) to the spermatozoa for their maturation and long-term viability. The aim of the present study was to investigate the distribution, classification, and source of multivesicular bodies (MVBs) and their EVs in the epithelia of the efferentes duct in a turtle species, the soft-shelled freshwater turtle *Pelodiscus sinensis* by using light and transmission electron microscopy. The results showed that CD63 as a classical exosome marker was strongly immunolocalized within the apical and lateral cytoplasm of the ciliated cells (CC) and moderate to weak in the non-ciliated cells (NCC) of DE. The ultrastructure revealed that early endosome was present at the basement membrane and perinuclear cytoplasm of both CC and NCC, whereas MVBs were located over the nucleus in the cytoplasm of NCC and adjacent to the basal bodies of cilia within the CC. Many EVs, as sources of MVBs, were located within the blebs that were attached to the cilia of CC, within the apical blebs from NCC, and the lateral spaces of CC and NCC. There was ultrastructure evidence of EVs associated with spermatozoa in the lumens of DE. Collectively, the present study provides cytological evidence that the DE epithelium secreted EVs to the lumen by (1) apical blebs, (2) ciliary blebs, and (3) from the basolateral region. These EVs were associated with spermatozoa in the DE lumen of this turtle. Characterization and cellular distribution of these EVs in the DE of a turtle may provide a study model to further investigate the transferring of micromolecules via EVs to the spermatozoa.

## 1. Introduction

The soft-shelled freshwater turtle, *Pelodiscus sinensis,* has a seasonal reproductive pattern in which animals undergo hibernation (December to April) after their reproductive phase (May to late October), and in later (October), show a massive release of spermatozoa into the epididymis. Subsequently, the spermatozoa travel via the ductuli efferentes (DE) into the epididymis and are stored there during the hibernation phase until the following mating season (June to August) [1,2]. The DE is the only ducts which act as channels for the passage of spermatozoa from the rete testis to the epididymis [3]. In the soft-shelled turtle, the DE has 22–28 tubules that originate from the rete testis and adjoin with the epididymis. These segments comprise ciliated and non-ciliated cells and are organized as pseudostratified columnar epithelia [4]. In mammals, during the transit phase of spermatozoa, nearly 95% of the seminiferous tubular fluid is reabsorbed to increase the concentration of luminal spermatozoa. This function is accomplished due to a well-developed endocytosis system within the non-ciliated epithelia. Ciliated cells participate in physical movement of the spermatozoa towards the epididymis [5,6].

Interestingly, the DE cilia have a rotational twist and a random beat. The primary function of these cilia is to stir the luminal fluids to ensure the reabsorption of homogenous fluid by non-ciliated cells [7]. Both DE and the epididymis create and maintain the luminal microenvironment that is optimal for sperm viability and function [8], by secreting and expressing various proteins. Therefore, in the DE, intercellular communication might be performed via extracellular vesicles (EVs). As reviewed by Gyorgy [9], the term EVs has been suggested for all populations of cell-derived vesicles. The major populations of these EVs include exosomes, micro-vesicles, and apoptotic bodies. In the past few years, research interest has mainly focused on two major types; the exosome and micro-vesicles [9]. The exosome (size 50–100 nm) is released after the fusion of multivesicular bodies with the plasma membrane into the extracellular milieu [10,11], while the micro-vesicles (size 100–1000 nm) are generated by the budding/ blebbing of the plasma membrane [9]. To avoid confusion, in our study we used the term EVs for both exosomes and micro-vesicles. To study these EVs, we monitored the presence of CD63, which is considered the most common extracellular vesicle marker and localized within the endosomal system such as in late endosomes/MVBs [12,13]. Transmission electron microscopy has also been valued for its capability to detect and characterize the EVs [14]. Yanagimachi and his colleagues provided the first electron microscope-based evidence of epididymosomes, an EV in the epididymis. These were detected as heterogenous membranous EVs of 20–100 nm diameter, secreted by principal cells using apocrine secretion into the intraluminal compartment [15]. In every cell, these EVs possessed a conserved structure and have been well documented [9,16]. However, the composition of these EVs reflects their origin and points to their possible physiological role and targeting properties [17,18]. Despite all these studies, no data are available about EVs in the ductuli efferentes.

An important aspect of DE is the expression of the hormonal receptors that are suggested to be necessary for sperm maturation and fertility. The fat sand rat, a seasonal reproductive animal, showed a reduction in immunoreactivity of androgen-estrogen receptors in epididymis after ligation of the efferentes duct [19]. While in rabbits, androgen binding protein is secreted in the DE and is suggested as a carrier of androgen from the testis to the epididymis [20]. Such receptors or proteins were transferred via EVs. Furthermore, the ductuli efferentes connect the rete testis to the epididymis, however, to best of our knowledge, till now there are no data documented regarding EVs source and their distribution and classification within the DE epithelial cells. Nevertheless, much attention has been paid to DE morphology and functional aspects in mammals and reptiles [4,21,22,23,24]. Hence, the present study was designed and investigated the EVs source and their distribution and classification in the DE epithelium, and their interaction with spermatozoa in the ductuli efferentes of a turtle.

## 2. Materials and Methods

### 2.1. Experiment Animals

*Pelodiscus sinensis* are soft-shelled turtles (5 mature males, >3 years age) that were purchased from an aquafarm in Nanjing, Jiangsu province of China, during late October to early November (a period of spermiation). Animals were treated in accordance with the guidance approved by the Science and Technology Agency of Jiangsu Province (SYXK (SU) 2010-0005). All the animals were rendered comatose using intraperitoneally administered sodium pentobarbital (20 mg/animal) and were sacrificed by cervical dislocation. The corpus segment of the epididymis was collected immediately and fixed to perform different techniques (details below).

### 2.2. Light Microscopy

To analyze the ductuli efferentes in the collected samples of the epididymis, samples were placed in 10% neutral buffered formalin for fixation overnight and then embedded in paraffin wax. Sectioning was done at 5 µm. These sections were stained with hematoxylin and eosin procedures (Harris’s hematoxylin for 2 min and 1% eosin for 30 s) for light microscopy analysis using an Olympus microscope (BX53), with a camera (Olympus DP73) (Olympus, Tokyo, Japan).

### 2.3. CD63 Immunohistochemistry

The paraffin sections prepared on glass slides were briefly deparaffinized and washed with phosphate buffer saline (PBS) ((P7059) Sigma-Aldrich, Darmstadt, Germany). Then, endogenous peroxidase was blocked and microwave antigen retrieval was undertaken with BSA (bovine serum albumin) (Boster Bio-Technology Co., LTD, Pleasonton, California, CA, USA) blocking for 1 h at 37 °C. After washing with PBS, the tissue was incubated overnight at 4 °C with a primary rabbit anti CD63 antibody (ab134045, Abcam, Cambridge, UK) (1:75 dilution). Then, sections were washed with PBS and were incubated for 30 min with biotinylated anti-rabbit antibody (KIT-5004/5/6) (MXB Biotechnology). Peroxidase was visualized with DAB (4′,6-diamidino-2-phenylindole) (Boster Bio-Technology Co., LTD, Pleasonton, California, CA, USA), and the sections were counterstained with hematoxylin. Sections incubated in PBS alone served as a negative control.

### 2.4. Transmission Electron Microscopy (TEM)

The collected samples of the middle epididymis were cut into small parts and fixed in 2.5% (v/v) glutaraldehyde ((G5882) Sigma-Aldrich, Darmstadt, Germany) in 0.1 M phosphate-buffered saline ((P7059) Sigma-Aldrich, Darmstadt, Germany) (PBS; 4 °C, pH 7.4; overnight). Thereafter, they were post-fixed with 1% (w/v) osmium tetroxide ((419494) Sigma-Aldrich, Darmstadt, Germany) in the buffer for 1 h and then washed in buffer. The samples were dehydrated in increasing concentrations of ethanol and infiltrated with a propylene oxide-Araldite mixture for embedding in Araldite ((A3183) Sigma-Aldrich, Darmstadt, Germany). The blocks were sectioned using an ultramicrotome (Reichert Jung, Vienna, Austria), and ultrathin sections (50 nm) were mounted on Formvar-coated grids and stained with uranyl acetate and Reynolds lead citrate for 20 min per step. The sections were analyzed with TEM (Hitachi H-7650; Tokyo, Japan).

## 3. Results

### 3.1. CD63 Immunohistochemistry of Ductuli Efferentes in Chinese Soft-Shelled Turtle

**Figure 1:**
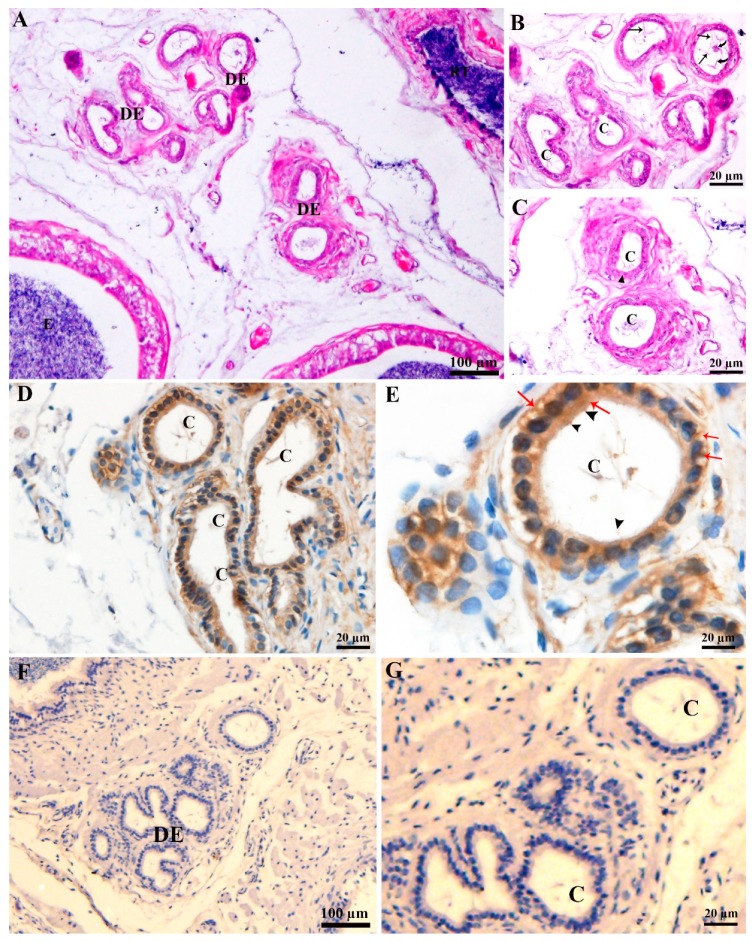
Light microscopy of ductuli efferentes. (**A**) Ductuli efferentes: a passage from rete testis to epididymis. (**B**,**C**) Cilia: a long protruding meshwork (black arrow) from ciliated cells with sperm (curved arrow) in the lumen of ductuli efferentes. (**D**,**E**) CD63 Immunohistochemistry (Redarrow showed strong labeling) in the ductuli efferentes. (**F**,**G**) PBS served as a negative control. C: cilia; DE: ductuli efferentes; E: epididymis; RT: rete testis;. Scale bar: A and F: 100 µm; B–E and F: 20 µm.

Light microscopic observation of DE in Chinese soft-shelled turtle (Figure 1 shows sections through DE), and sperm were exhibited in the DE lumens (Figure 1A). The detailed observation indicates that DE had distinct populations of ciliated cells (CC) and non-ciliated cells (NCC). The nuclei of CC and NCC are located apically and basally, respectively. Numbers of cilias originating from the CC into the lumen of DE, forming a network that had spermatozoa (Figure 1B,C). The CD63 immunohistochemistry results indicated moderate to strong immunosignals, mainly observed within the apical cytoplasm of CC. More precisely, the strong CD63 immunopositivity was observed within the apical region where cilia protrude out to the lumen of the DE. Between CC and NCC, the CD63 immunolabeling was moderate to strong. Whereas, the cytoplasm of NCC showed weak to moderate localization of CD63 (Figure 1D,E).

### 3.2. Ultrastructure of Ductuli Efferentes and Distribution of Early Endosomes and Multivesicular Bodies

The morphology of DE consists of the CC and NCC as described earlier by our research group [4]. The nuclei of CC were ovoid, and mitochondria were predominantly observed in the perinuclear cytoplasm. Coated pits and junctional complexes were frequently observed at apical borders of CC and vesicles were evenly distributed throughout the cytoplasm in the CC and NCC. The nuclei of NCC were euchromatic and mitochondria were mainly observed in the perinuclear and supranuclear cytoplasm. The lumen of ductuli efferentes showed various microvilli and few cilia. (Figure 1A,B).

**Figure 2:**
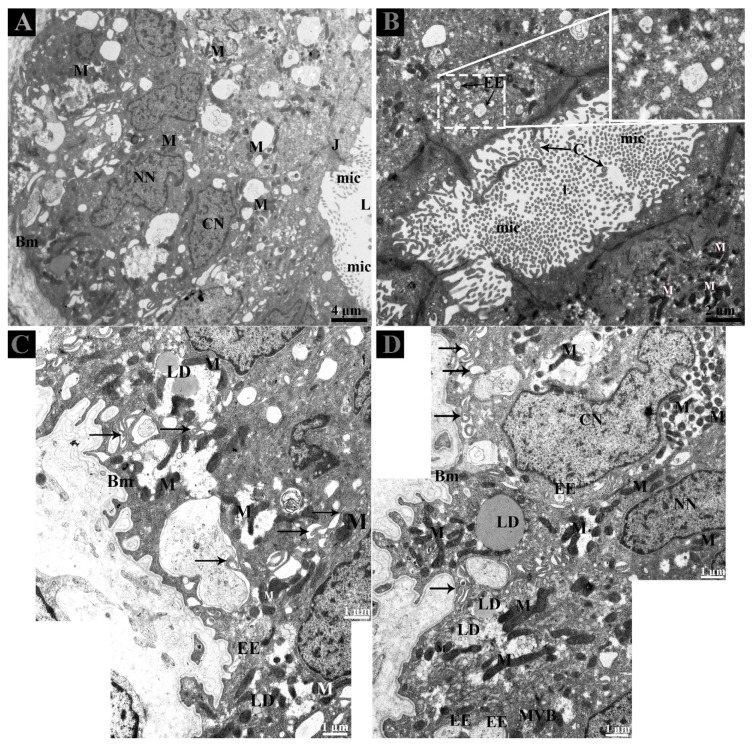
Ultrastructure of ductuli efferentes in Chinese soft-shelled turtle. (**A**) Ductulus efferentes comprises ciliated and non-ciliated cells and (**B**) lumen shows various microvilli and cilia. (**C**,**D**) At the basal membrane, early endosomes and multivesicular bodies were present adjacent to the lipid droplets and interdigitations (black arrow). BM: basement membrane; C: cilia; CN: ciliated cells nuclei; EE: early endosome; L: lumen; LD; lipid droplet; M: mitochondria; Mic: microvilli; MVB; multivesicular body; NN: non-ciliated cells nuclei. Scale bar: A: 4 µm; B: 2 µm; C–D: 1 µm.

**Figure 3:**
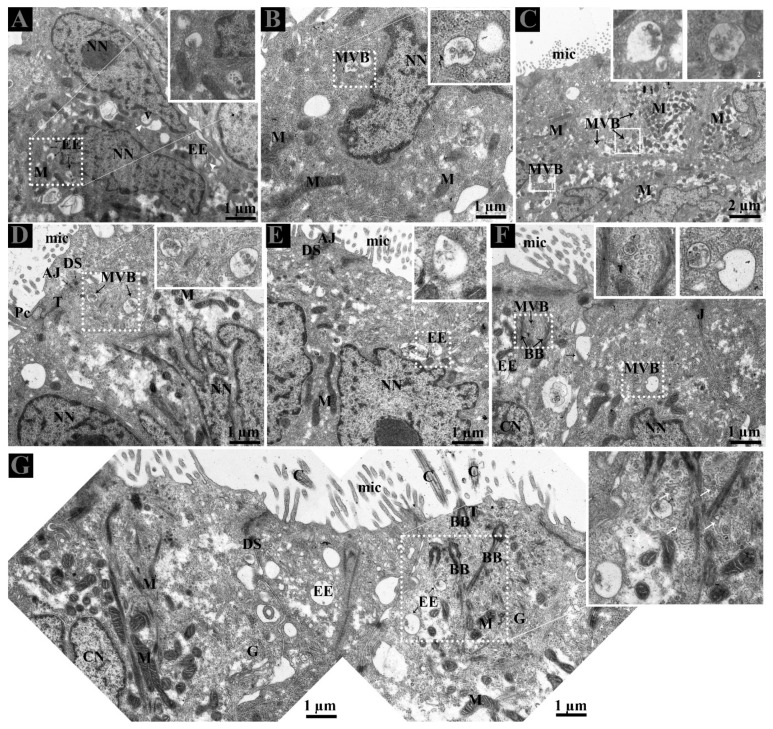
Distribution of early endosome and multivesicular bodies in ductuli efferentes. (**A**–**G**) EE and MVB located supra and perinuclear region of non-ciliated cells and (**F**,**G**) with small vesicles (white arrow) at the basal body of cilia (black arrow) in the ciliated cells. White arrowhead: interdigitations. White dotted rectangular area: enlarged area. AJ: adherent junction; Bb: basal body; C: cilia; CN: ciliated cells nuclei; DS: desmosomes; EE: early endosome; G: Golgi apparatus; M: mitochondria; Mic: microvilli; MVB; multivesicular body; NN: non-ciliated cells nuclei; T: tight junction. Scale bar: A–B and D–G: 1 µm; C: 2 µm.

Careful observation of the basal region of DE showed that lipid droplets and mitochondria exist near interdigitations and cytoplasmic spaces of CC and NCC. Fascinatingly, early endosomes (EE) were also clearly observed with heterogeneity of few or more intraluminal vesicles (ILVs) (Figure 2C,D). In the DE middle region, the EE and multivesicular bodies (MVBs) were observed within the peripheral cytoplasm of NCC and near the interdigitations (Figure 3A–E). EE, MVBs with varied numbers of ILVs (Intra-Luminal Vesicles), and numerous small vesicles were observed at the basal body of cilia from the CC (Figure 3F,G).

### 3.3. Extracellular Vesicles and Their Interaction with Spermatozoa in the Lumen of Ductuli Efferentes

**Figure 4:**
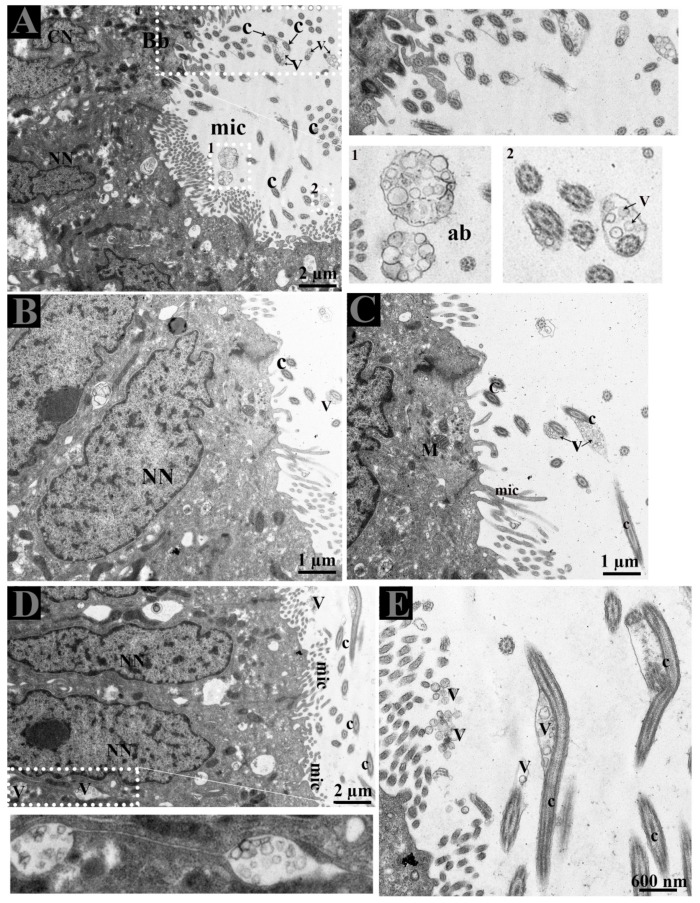
Extracellular vesicles and spermatozoa. (**A**–**C**) Extracellular vesicles in apical blebs from non-ciliated cells, in blebs with cilia from the ciliated cells, (**D**) in the lateral distinct boundary between cells and (**E**) in the lumen. The white dotted rectangular area shows an enlarged area. Ab: apical blebs; C: cilia; CN: ciliated cell nuclei; EE: early endosome; Mic: microvilli; MVB; multivesicular body; NN: non-ciliated cell nuclei; V: vesicles. Scale bar: A and D: 2 µm; B–C: 1 µm; E: 600nm.

**Figure 5:**
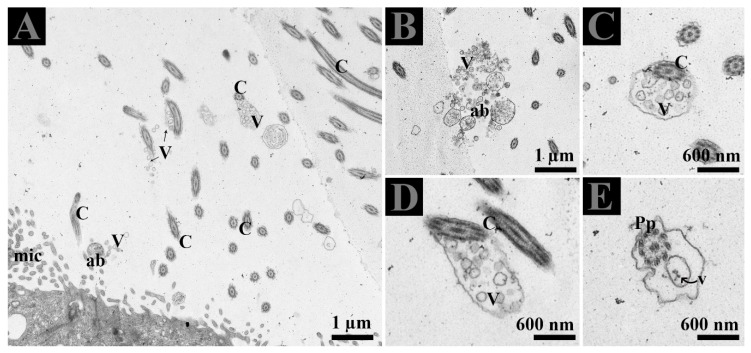
(**A**–**E**) Ultrastructure of extracellular vesicles and the principal piece of spermatozoa within the lumen of DE. Ab: apical blebs; C: cilia Mic: microvilli: Pp: principal piece of spermatozoa; V: vesicles. Scale bar: A–B: 1 µm; C–E 600 nm.

The lumen of DE showed microvilli, apical blebs, and cilia. Microvilli and cilia can easily be distinguished due to their thickness and morphological appearance. Apical blebs were observed over the microvilli from the NCC (Figure 4A). However, cilia showed blebs containing numerous vesicles that became more numerous with the size of blebs, for that reason, we suggest using the name ciliary blebs (Figure 4A,B). Many extracellular vesicles (EVs) appeared in the lumen and between the distinct lateral boundary of CC and NCC. Collectively, the DE epithelia possessed a well-developed exocytic pathway and showed numerous EVs that might be secreted by the shedding of apical blebs or ciliary blebs. Hence, EVs in DE originate in a different way. In addition, at the ultrastructural level, these EVs are associated with the principal piece of the spermatozoa (Figure 5E). 

## 4. Discussion

The ductuli efferentes (DE) provides an indispensable connection between the testis and epididymis and their normal function is required for male fertility [7]. In *Pelodiscus sinensis*, the morphology of DE was found to be similar to that described previously in a turtle, a crocodile, and a lizard [25,26,27]. Within the DE, the transport of spermatozoa required the assistance of cilia from the ciliated cells (CC) using a rotational twist and an arbitrary beat to ensure their viability in the luminal microenvironment. While non-ciliated cells (NCC) reabsorb the homogenous fluid for higher concentration of spermatozoa [7]. Cell-cell communication is a very complicated mechanism for information exchange and is adapted by direct or indirect contact of cells or via the release of EVs to transfer essential information to their target cells [28,29]. In the present study, the EVs originate in different ways from the DE epithelia and that associates with the spermatozoa in the lumen of DE. We used TEM and CD63 immunohistochemistry to study the EVs, their static cellular evidence of different events in the EVs maturation, and modes of secretion by the epithelium of DE in the turtle.

The biogenesis of EVs, such as exosomes, involve principle components including endosomal machinery generated early endosomes that mature into late endosomes or multivesicular bodies (MVBs). They are released into the extracellular space after MVBs fuse with the plasma membrane. The enclosed intraluminal vesicles (then referred to as exosomes) are released into the extracellular space for communication [30]. The release of such EVs is an interesting fact; many studies suggest that the apical and basolateral region of polarized cells differ in composition, thus supporting the concept that different MVBs populations originate from different cellular locations [31,32,33,34]. In our study, early endosomes were observed at the basement membrane and at the perinuclear cytoplasm of CC and NCC, whereas MVBs were located at supra nuclear cytoplasm of NCC and adjacent to the basal body of cilia in the CC. Interestingly, our results also showed numerous EVs present at lateral distinct spaces between CC and NCC of the DE (Figure 4D). In proximal tubular epithelial cells of the kidney, the site of EVs (exosome) release were compared in-vivo and suggested that those EVs secreted apically were greater in numbers than from the basolateral region. Additionally, the CD63 was expressed at greater than a fourfold level in apically secreted exosomes than in the basolateral region [35]. Fascinatingly in our study, the CC showed strong CD63 expression apically as compared to moderately in the lateral sides, whereas NCC expression was only moderate to weak. Collectively, the combined use of TEM and CD63 immuno-expression supports the view that CC and NCC possessed well developed EV exocytosis systems.

Epithelial cells of the male reproductive system involved in apocrine secretion have been well-studied in the epididymis and prostate. The small membranous vesicles in apical blebs are released into the extracellular milieu by apocrine secretion [29,36,37,38,39]. Apocrine secretion is characterized by the formation of apical blebs by the epithelium, where the apical blebs detach from the apical membrane and then release their contents into the lumen through fragmentation [29,36,38]. A similar mode of apocrine secretion in the epididymis of the Chinese soft-shelled turtle was documented [40]. Moreover, in previous studies and in the ultrastructure findings of the present study (Figure 4 and Figure 5), it is concluded that NCC actively form and secrete apical bleb secretions and release their contents into the extracellular milieu of DE. Furthermore, early endosome and MVB occur at peri and supranuclear regions and are subsequently found within the apical blebs. We believe that NCC contain a well developed exocytotic system and apical bleb served as a vehicle to discharge the EVs into the luminal environment to interact with sperm in the DE. The well-developed apical bleb secretion by NCC in the DE is proposed to be involved in sensing the fluid to modify its ion and protein concentrations and composition, that will subsequently be homogenized by fluid phase processes [41]. Besides that, our study also revealed cilia bleb secretion by the CC. The existence of numerous vesicles adjacent to the basal body of the cilia and the developing bleb with numerous vesicles attached to the cilia were also observed (Figure 4). Cocucci has explained the shedding of such vesicles by budding off small cytoplasmic protrusions [29]. The secretion of such developing blebs attached to the cilia has not been previously reported. Therefore, the vesicles inside such developing blebs, assisted by the cilia, are also a novel observation in the CC of the turtle DE. Moreover, our study also provides TEM evidence of EVs that attach to the principal piece of spermatozoa in the DE (Figure 5E). Therefore, collectively it can be speculated that in DE, these EVs provide an assisting role to the spermatozoa for their viability and could be delivering molecules such as RNA, which has no impact on viability but may have an influence on offspring by direct information transfer, as it is a very important aspect of communication [42]. Moreover, in mammals, it is reported that DE fluid possessed a high affinity of androgen binding protein carried into the epididymis [20]. While induced abnormalities at a neonatal age resulted in DE lowered epithelial cell height and imbalance in androgen-estrogen level [43]. Hence, it is reasonable to suggest that EVs secreted by the DE play a vehicle role to transfer RNA as well as proteins for sperm maturation within the epididymis and as stated above, to influence the offspring. Where these EVs came from apical blebs of NCC or were secreted from cilia blebs or the lateral side from the CC into the DE luminal milieu are still a question. Meanwhile, these EVs inside the lumen of DE expand and open the next door of research into what kind of information exchange occur between DE secreted EVs and spermatozoa? 

## 5. Conclusions

**Figure 6:**
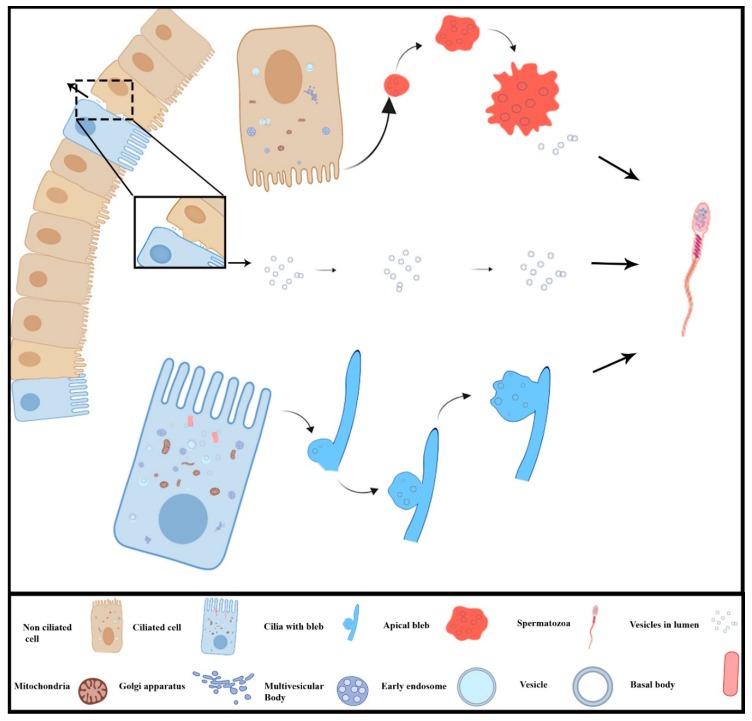
Schematic diagram of different routes of micro-vesicles secreted by the ciliated and non-ciliated cells of ductuli efferentes in the turtle.

In conclusion, the present study investigated for the first time the EVs mode of secretion in the DE epithelium of turtle. The ciliated cells secrete EVs apically through the cilia blebs while non-ciliated cells do so by the apical blebs, both into the DE luminal microenvironment. More importantly, DE epithelium also secretes EVs via the basolateral region. Additionally, these EVs are associated with spermatozoa in the lumen of DE. An illustration of these different routes of EVs secreted by epithelia of ductuli efferentes was presented in Figure 6. The present study provides the EVs secretion by the ciliated and non-ciliated cells and their interaction with the spermatozoa in the DE lumen of the *Pelodiscus sinensis* turtle.
